# A Phase I Study of Ribociclib Plus Everolimus in Patients with Metastatic Pancreatic Adenocarcinoma Refractory to Chemotherapy

**DOI:** 10.1089/pancan.2020.0005

**Published:** 2020-06-22

**Authors:** Benjamin A. Weinberg, Hongkun Wang, Agnieszka K. Witkiewicz, John L. Marshall, Aiwu R. He, Paris Vail, Erik S. Knudsen, Michael J. Pishvaian

**Affiliations:** ^1^ Ruesch Center for the Cure of Gastrointestinal Cancers, Lombardi Comprehensive Cancer Center, Georgetown University Medical Center, Washington, District of Columbia, USA.; ^2^ Roswell Park Cancer Institute, Buffalo, New York, USA.; ^3^ Sidney Kimmel Comprehensive Cancer Center at Sibley Memorial Hospital, Johns Hopkins University School of Medicine, Washington, District of Columbia, USA.

**Keywords:** CDK4/6, mTOR, ribociclib, everolimus, pancreatic adenocarcinoma

## Abstract

**Purpose:** Metastatic pancreatic adenocarcinoma (mPC) has a poor prognosis. CDK4/6 is often deregulated in mPC due to *CDKN2A* loss, resulting in the loss of p16INK4a that inhibits CDK4/6. CDK4/6 inhibitor monotherapy is ineffective due to RAS-mediated activation of alternative pathways, including phosphatidylinositol 3-kinase–mammalian target of rapamycin (PI3K-mTOR). We conducted a phase I study combining CDK4/6 and mTOR inhibition in patients with mPC refractory to standard chemotherapy.

**Materials and Methods:** The combination of ribociclib (a CDK4/6 inhibitor) and everolimus (an mTOR inhibitor) was investigated in a phase I study in patients with mPC and progression on 5-fluorouracil- and gemcitabine-based chemotherapy. A 3 + 3 design was used to find the recommended phase II dose (RP2D) of ribociclib (250 or 300 mg daily for days 1–21) in combination with everolimus (2.5 mg daily for days 1–28) every 28 days. Secondary endpoints were median progression-free survival (mPFS), median overall survival (mOS), response rate, safety, and effect on the retinoblastoma pathway.

**Results:** Twelve patients were enrolled, six at each dose level. Only one patient had a dose-limiting toxicity of a grade 3 rash at the 250 mg dose. The RP2D of ribociclib was 300 mg. mPFS was 1.8 months (95% confidence interval [CI] [0.6–2.1]), and mOS was 3.7 months (95% CI [2.3–5.6]). Two patients (17%) had stable disease at 8 weeks. Pharmacodynamic evaluation demonstrated that CDK4/6-regulated gene expression was significantly decreased on treatment (*n* = 6, *p* < 0.001).

**Conclusion:** Ribociclib 300 mg daily for days 1–21 plus everolimus 2.5 mg daily was well tolerated and associated with decreased CDK4/6-regulated gene expression. This combination was not effective as a third-line therapy but does pharmacologically target CDK4/6 in mPC, revealing the potential for benefit in other settings.

## Introduction

Cancer of the pancreas is the third leading cause of cancer deaths in the United States, with an estimated 57,600 new diagnoses and 47,050 deaths attributable to the disease in 2020.^1^ Surgical resection offers the only chance of cure for pancreatic adenocarcinoma (PC). However, only 15–20% of patients have resectable disease at initial diagnosis; the majority have either locally advanced or metastatic cancer (metastatic pancreatic adenocarcinoma [mPC]). Of those who are surgical candidates, most will have disease relapse after surgery. Five-year survival rates are only 37% for those diagnosed with localized disease and 3% for those diagnosed with metastatic disease. Novel therapies are desperately needed.

Cell cycle progression is a tightly regulated process, and aberrant cell cycle regulation is a hallmark of most cancers.^2^ G1 to S-phase progression is regulated by the cyclins and cyclin-dependent kinases (CDKs). CDK4 and its closely related homolog, CDK6, are activated by the D-type cyclins, enabling progression in the early G1 phase. CDK2 is activated by the A- and E-type cyclins, enabling progression through late G1 and into S-phase.^2^ CDK4/6 and CDK2 activities are inhibited by the CDK inhibitors, p16^INK4^ and p21^Cip/Kip^, respectively. CDK4/6 and other CDKs and cyclins are often overexpressed in tumor cells, and activating mutations in CDK4 are oncogenic.^2–10^ CDK4 and CDK6 classically exert their effects on the cell cycle by phosphorylating retinoblastoma (RB) protein, enabling the release of the E2F transcription factor, resulting in the transcription of genes required for subsequent cell cycle events, such as cyclins E and A.^2^ However, data demonstrate that CDK4/6-regulated cell cycle activation also occurs through the inhibition of TGF-β signaling.^11^

The activity of CDK4/6 is frequently upregulated in PC due to the loss of *CDKN2A* through either homozygous deletion or epigenetic silencing. The signature driver of PC (Kirsten rat sarcoma virus gene [KRAS]) propels cells into senescence through activation of CDKN2A. Thus, there is a potent hypothetical rationale for pharmaceutically mimicking the function of CDKN2A based on a plethora of published data. In the sequencing of over 109 cases, we discovered *CDKN2A* loss (41%) and amplification of CCND1 (9%) and CDK4 (6%), whereas the loss of RB is rare (3%).^12^ Therefore, from a genetic perspective, one would anticipate that PC could respond efficiently to CDK4/6 inhibition.

**FIG. 1.**
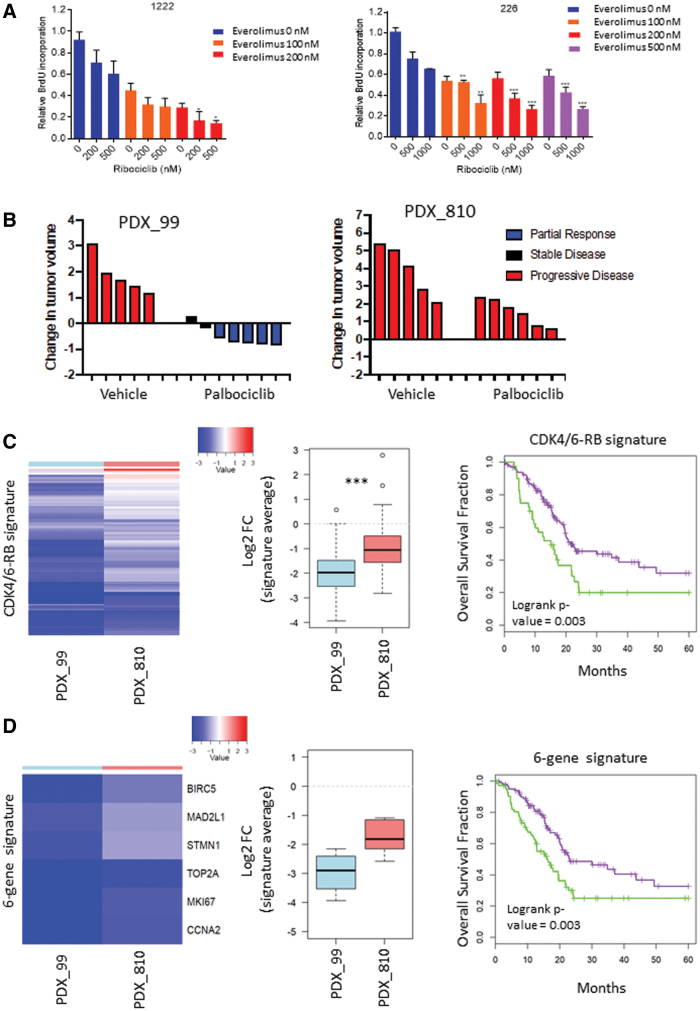
Preclinical modeling of combined CDK4/6 and mTOR inhibition in mPC. **(A)** The indicated PC cell lines were treated with ribociclib and everolimus at the indicated concentrations for 48 h and bromodeoxyuridine incorporation was determined (*p < 0.05, **p < 0.01, ***p < 0.001). Drug synergy was determined with synergy-finder software. **(B)** The indicated PDX models were treated with vehicle or palbociclib for 21 days and the change in tumor volume was determined. The partial response or progressive disease was determined with a 30% change in tumor volume during the course of treatment. **(C)** RNA sequencing of the indicated PDX models was used to investigate the CDK4/6-RB signature. Heatmap depicts 182 genes that are summarized in the box plots (***p < 0.001). This same gene expression signature was applied to the TCGA pancreatic cancer data set. **(D)** RNA sequencing of the indicated PDX models was used to investigate a subset of six genes within the CDK4/6-RB signature. Heatmap depicts the six genes that are summarized in the box plots (*p* = 0.14). This same gene expression signature was applied to the TCGA pancreatic cancer data set. mPC, metastatic pancreatic adenocarcinoma; mTOR, mammalian target of rapamycin; PDX, patient-derived xenograft; RB, retinoblastoma; TCGA, The Cancer Genome Atlas.

Treatment with CDK4/6 inhibitors can lead to an accumulation or maintenance of mammalian target of rapamycin (mTOR) activity as measured by phosphorylated S6 kinase. Although phosphatidylinositol 3-kinase (PI3K) inhibitors have only modest effects, mTOR inhibitors are synergistic when combined with CDK4/6 inhibitors for the suppression of proliferation and active cell killing (Fig. 1). In contrast, a component of the mitogen-activated protein kinase pathway inhibitors suppress proliferation but do not induce apoptosis in these models. Identical results were observed by independent groups using different cell lines and xenograft models.^13,14^

Given the upregulation of CDK4/6 and mTOR signaling shown in gene analysis of PC, inhibition of PC by CDK4/6 inhibitor in xenograft models, and increase in sensitivity of PC to CDK4/6 inhibition by mTOR inhibitors, there is a strong rationale to combine CDK4/6 and mTOR inhibitors in PC treatment. The use of combination approaches is particularly important because resistance to CDK4/6 inhibitor monotherapy can develop in PC models, and data reveal that mTOR inhibitors are particularly effective at countering the acquisition of resistance and actively killing cells treated with CDK4/6 inhibitors.^13,14^

Ribociclib (Novartis Pharmaceuticals Corporation) is an oral CDK4/6 inhibitor, U.S. Food and Drug Administration (FDA) approved for postmenopausal women with advanced breast cancer that is hormone receptor positive and HER2 negative, in combination with an aromatase inhibitor.^15^ Everolimus (Novartis Pharmaceuticals Corporation) is an oral mTOR inhibitor, FDA approved for patients with metastatic breast cancer, neuroendocrine tumors, renal angiomyolipoma, tuberous sclerosis complex, and renal cell carcinoma.^16^

The combination of ribociclib, everolimus, and exemestane was previously tested in a phase Ib study (NCT01857193). Everolimus was administered at 2.5 mg daily concurrently with 200, 250, and 300 mg of ribociclib (daily, days 1–21 every 28 days) with or without food. In addition, 350 mg of ribociclib was administered with 1 mg of everolimus without food or 2.5 mg everolimus with food and 200 mg of ribociclib was administered with 5 mg of everolimus with food. A fixed dose of 25 mg of exemestane daily was administered in each combination.^17^ Further evaluation of 2.5 mg of everolimus daily with 300 mg ribociclib for days 1–21 every 28 days with food is ongoing in dose expansion.^17^

Here we report the results of a phase I study of ribociclib and everolimus in patients with chemorefractory mPC (NCT02985125).

## Materials and Methods

### Study design and objectives

This was an open-label single-arm single-institution dose-escalation phase I study of ribociclib and everolimus in patients with mPC. The primary objective was to determine the recommended phase II dose (RP2D) and schedule of ribociclib in patients treated with the combination based on the maximum tolerated dose. Secondary objectives included determination of safety and tolerability, median progression-free survival (mPFS), median overall survival (mOS), best overall response rate, best change in serum tumor marker (CA 19-9), and the pharmacodynamic effects of treatment on the RB pathway.

### Study treatment

Patients were enrolled in cohorts of three patients each following a standard 3 + 3 design. Patients enrolled at dose level 1 received ribociclib 250 mg daily for days 1–21 and everolimus 2.5 mg daily for days 1–28 every 28 days, and patients enrolled at dose level 2 received ribociclib 300 mg daily for days 1–21 and everolimus 2.5 mg daily for days 1–28. Two potential dose de-escalation levels (level-1: ribociclib 200 mg daily for days 1–21, level-2: ribociclib 150 mg daily for days 1–21) were included in the protocol but were not reached. The everolimus 2.5 mg daily dose was based on the pharmacokinetics data already described.

### Patient population

Patients with mPC and prior disease progression on both 5-fluorouracil- and gemcitabine-based chemotherapy regimens for advanced disease were eligible for this study. Development of metastatic disease during or within 6 months after completion of adjuvant chemotherapy counted as disease progression on that chemotherapy regimen. Inclusion criteria included histologically confirmed PC with measurable disease amenable to serial biopsy, age at least 18 years, Eastern Cooperative Oncology Group (ECOG) performance status of 0–2, adequate bone marrow and hepatorenal function, QTcF <450 ms, and ability to provide informed consent.

### Safety and efficacy assessments

Patients were assessed for the development of adverse events using the Common Terminology Criteria for Adverse Events (CTCAE) v 4.03. Predefined dose-limiting toxicities (DLTs) were determined during the first treatment cycle (28 days, Supplementary Table S1). Tumor assessments occurred during screening and every 8 weeks with computed tomography or magnetic resonance imaging scans, and tumor response was assessed using response evaluation criteria in solid tumors (RECIST) v. 1.1 as determined by the local investigator.

### Biomarker assessments

Patients underwent a pretreatment tumor biopsy during screening and an on-treatment biopsy at cycle 1 on day 15. Archival tissue was not allowed. Tumor biopsies were fixed in formalin and paraffin embedded. These samples underwent targeted next-generation sequencing of 196 genes and immunohistochemical testing for RB pathway marker expression including RB, pRB, p16, cyclin D1, Ki67, MCM7, and pS6.

### PDX 99/810 comparison

RNA sequencing was performed on triplicate PDX models for control and palbociclib treated samples.^18^ RNA sequencing counts were normalized and log-transformed using the edgeR R package. Log-fold changes (logFC) were calculated by comparing mean palbociclib-treated sample expression to mean control sample expression. CDK4/6 signature genes, or selected subset, were extracted and compared.

### TCGA survival

TCGA expression and survival data was downloaded through the portal. Median normalized expression was log-transformed and CDK4/6 signature genes, or selected subset, were extracted. Samples were clustered into two groups using hierarchical clustering and survival analysis was performed using the survival package in R.

### NeoPalAna comparison

The six patients with pre- and post-treatment biopsies were compared to the NeoPalAna ER+ breast cancer patients.^19^ LogFCs were calculated for samples with matched baseline and C1D15 treatment samples. CDK4/6 signature genes, or selected subset, were extracted and compared.

### Statistical analyses

Patients' characteristics and adverse events were collected and tabulated using descriptive statistics. Pharmacodynamic comparisons in pre- versus on-treatment tumor samples were performed using paired student's *t*-test, and survival outcomes were estimated using the Kaplan–Meier method.

### Ethics

This clinical trial protocol and informed consent form were approved by the institutional Clinical Review Committee and Institutional Review Board at Georgetown University. The study was conducted according to the International Committee on Harmonization Guidelines for Good Clinical Practice and the ethical principles outlined in the Declaration of Helsinki. All patients provided written informed consent before screening for the study.

## Results

Twelve patients were enrolled between May 2017 and May 2019. The median age of accrued subjects was 68.4 years (range 43.9–77.3 years), 10 patients were women, and 58% had an ECOG performance status of 1 (Table 1). Most patients (83%) had received two or more prior lines of chemotherapy for mPC, and 67% had two or more sites of metastatic disease.

**Table 1.** Patient Characteristics

Characteristic	Ribociclib 250 mg daily for days 1–21 plus everolimus 2.5 mg daily for days 1–28 n = 6	Ribociclib 300 mg daily for days 1–21 plus everolimus 2.5 mg daily for days 1–28 n = 6
Median age, years (range)	55.1 (43.9–77.3)	69.8 (59.2–72.0)
Male, *n* (%)	1 (17)	1 (17)
ECOG PS		
0	2 (33)	2 (33)
1	3 (50)	4 (67)
2	1 (17)	0 (0)
Prior surgery, *n* (%)	2 (33)	4 (67)
No. of prior regimens, *n* (%)		
1^a^	1 (17)	1 (17)
2	3 (50)	5 (83)
3 or greater	2 (33)	0 (0)
No. of metastatic sites, *n* (%)		
1	2 (33)	2 (33)
2	2 (33)	2 (33)
3	2 (33)	2 (33)
Sites of metastatic disease, *n* (%)		
Liver	5 (83)	4 (67)
Lung	3 (50)	5 (83)
Peritoneum	3 (50)	3 (50)
Bone	1 (17)	1 (17)
Baseline CA19-9, average (range)	4652.8 (0–18038.6)	8358.4 (108.8–17424.7)

^a^ Two patients had neoadjuvant/adjuvant chemotherapy that counted as prior regimens.

ECOG PS, Eastern Cooperative Oncology Group Performance Status.

### Study treatment

Six patients were enrolled at dose level 1 and six were enrolled at dose level 2. One patient at dose level 1 came off study due to a DLT at cycle 1 on day 16, and all other patients came off study for disease progression, including one patient who progressed on imaging during her cycle 1 on day 15 tumor biopsy. Both of these patients who came off study during cycle 1 were still assessed for DLTs. Median time on study was 55 days (range 16–91 days).

### Dose-limiting toxicities

The one patient who experienced a DLT developed a grade 3 maculopapular rash, which was thought to be an allergic reaction. No other DLTs were observed. Six patients each were enrolled in dose levels 1 and 2, and when administered with 2.5 mg everolimus daily, the RP2D of ribociclib was determined to be 300 mg daily on days 1–21 of each 28-day cycle.

### Safety and tolerability

All 12 patients were evaluable for safety analysis, and all patients experienced at least one adverse event (Table 2). Fifty-eight percent of patients experienced a grade 3/4 adverse event, 54% of which were hematological toxicities. The nonhematological grade 3/4 events that occurred were abdominal pain, diarrhea, rash, fever, dehydration, and sepsis (one each). One patient died while on study due to disease progression, not attributed to study drugs. There was no evidence of QTc prolongation in this study.

**Table 2.** All Adverse Events

	Ribociclib 250 mg daily for days 1–21 plus everolimus 2.5 mg daily for days 1–28 n = 6		Ribociclib 300 mg daily for days 1–21 plus everolimus 2.5 mg daily for days 1–28 n = 6	
AEs, *n* (%)	All grade	Grade 3/4	All grade	Grade 3/4
All AEs	6 (100)	3 (50)	6 (100)	4 (67)
Neutropenia	3 (50)	2 (33)	4 (67)	1 (17)
Lymphopenia	0 (0)	0 (0)	1 (17)	1 (17)
Anemia	1 (17)	1 (17)	2 (33)	1 (17)
Thrombocytopenia	1 (17)	0 (0)	4 (67)	1 (17)
Abdominal pain	2 (33)	0 (0)	1 (17)	1 (17)
Peripheral edema	1 (17)	0 (0)	1 (17)	0 (0)
Fracture	1 (17)	0 (0)	0 (0)	0 (0)
Nausea	2 (33)	0 (0)	1 (17)	0 (0)
Vomiting	2 (33)	0 (0)	0 (0)	0 (0)
Pruritus	1 (17)	0 (0)	0 (0)	0 (0)
Rash	2 (33)	1 (17)	0 (0)	0 (0)
Anorexia	1 (17)	0 (0)	0 (0)	0 (0)
Diarrhea	2 (33)	1 (17)	1 (17)	0 (0)
Dysgeusia	1 (17)	0 (0)	0 (0)	0 (0)
Fatigue	1 (17)	0 (0)	2 (33)	0 (0)
Oral mucositis	1 (17)	0 (0)	2 (33)	0 (0)
Epistaxis	1 (17)	0 (0)	0 (0)	0 (0)
Weight loss	1 (17)	0 (0)	1 (17)	0 (0)
Rectal hemorrhage	1 (17)	0 (0)	0 (0)	0 (0)
Urinary frequency	1 (17)	0 (0)	0 (0)	0 (0)
Acute kidney injury	1 (17)	0 (0)	2 (33)	0 (0)
Allergic rhinitis	0 (0)	0 (0)	1 (17)	0 (0)
Hypophosphatemia	0 (0)	0 (0)	1 (17)	0 (0)
AST increased	0 (0)	0 (0)	1 (17)	0 (0)
Chest wall pain	0 (0)	0 (0)	1 (17)	0 (0)
Neck pain	0 (0)	0 (0)	1 (17)	0 (0)
Fever	0 (0)	0 (0)	2 (33)	1 (17)
Dehydration	0 (0)	0 (0)	1 (17)	1 (17)
Hyperglycemia	0 (0)	0 (0)	1 (17)	0 (0)
Hyperkalemia	0 (0)	0 (0)	1 (17)	0 (0)
Sepsis	0 (0)	0 (0)	1 (17)	1 (17)
Infection	0 (0)	0 (0)	1 (17)	0 (0)
Sinus tachycardia	0 (0)	0 (0)	1 (17)	0 (0)

AEs, adverse events; AST, aspartate aminotransferase.

### Clinical activity

**FIG. 2.**
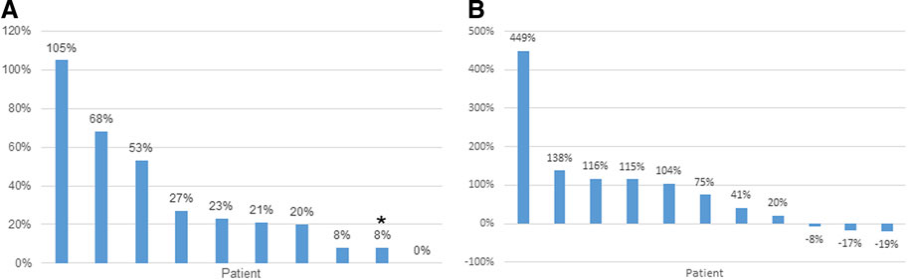
Tumor response. **(A)** The best overall response by RECIST v. 1.1. *New lesion. One patient had progression of disease but was not evaluable by RECIST v. 1.1, and one patient was not evaluable due to dose-limiting toxicity before tumor reassessment. **(B)** The best change in serum CA19-9. One patient had a tumor without positive CA19-9. RECIST, response evaluation criteria in solid tumors.

**FIG. 3.**
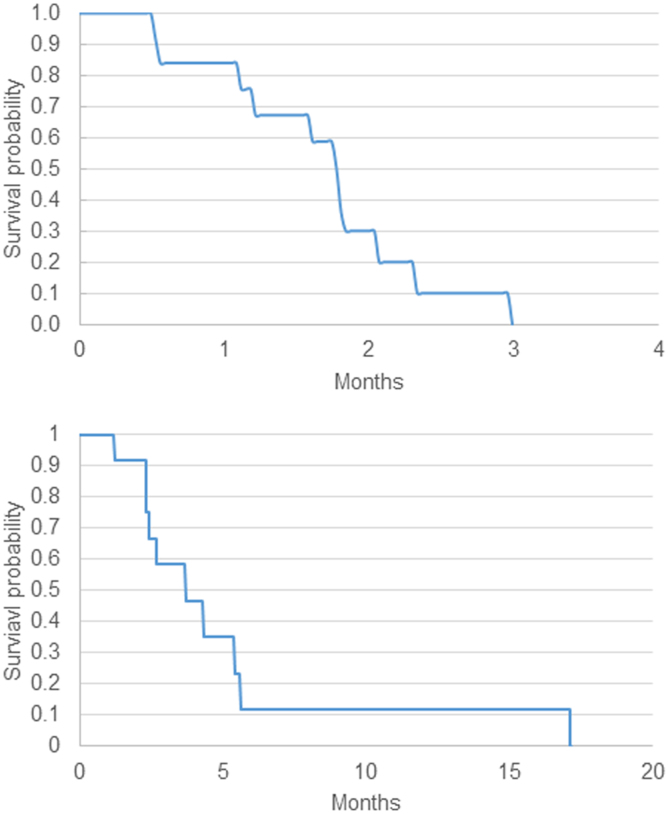
Kaplan–Meier estimates of progression-free and overall survival.

Eleven of the 12 patients were evaluable for response by RECIST v. 1.1. Two patients had stable disease at the first response assessment at 8 weeks, whereas nine others came off study for disease progression by 8 weeks (Fig. 2A). Three patients (27%) had an initial reduction in serum CA19-9 (Fig. 2B). mPFS was 1.8 months (95% confidence interval [CI] [0.6–2.1]) and mOS was 3.7 months (95% CI [2.3–5.6], Fig. 3). One patient was lost to follow up after 3 months, and one patient is still alive off study. Patient 11 had prolonged OS of 17 months despite having disease progression on trial. Interestingly, this patient's tumor next-generation sequencing analysis showed a pathogenic mutation in *KRAS* G12R (seen in ∼20% of PC), which may have an impaired ability to activate PI3K-mTOR signaling.^20^

### Pharmacodynamic analyses

**FIG. 4.**
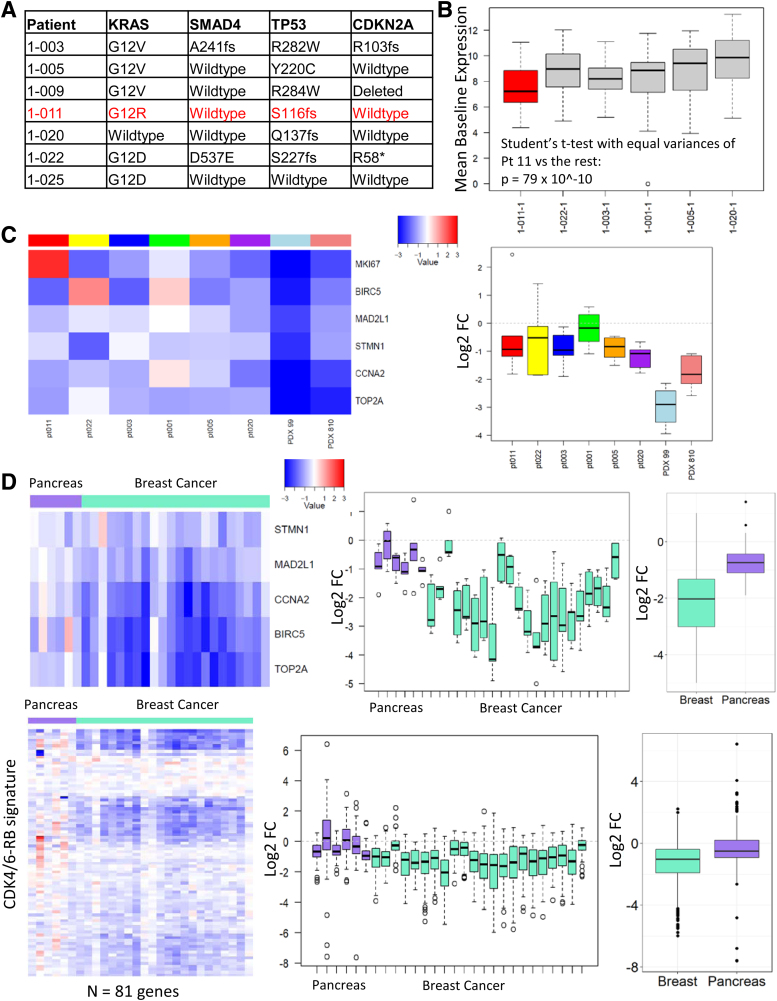
Pharmacodynamic effect of combined CDK4/6 and mTOR inhibition. **(A)** The canonical genomic alterations present in a subset of patients on the study. Patient no. 11 (highlighted in red) had the best response on study. **(B)** The average CDK4/6-RB signature in each of the six cases subjected to HTG Molecular Diagnostics, Inc. oncology panel. Patient no. 11 expresses significantly lower levels of this proliferation signature. **(C)** Benchmarking of transcriptional response from the patients on study versus the PDX models. Although there is a significant transcriptional repression in all PC cases, it is less than that observed in the model that progresses on treatment with palbociclib. **(D)** Benchmarking of the transcriptional response to everolimus and ribociclib versus breast cancer patients on the NeoPalAna trial who were receiving palbociclib and anastrozole. MKI67 was not sequenced as part of the NeoPalAna trial and was excluded from this analysis. There was a significant (>50% on average) decrease in expression of CDK4/6-regulated genes (e.g., *BIRC5*, *CCNA2*, *STMN1*, and TOP*2A*) in pre- versus post-treatment tumor samples (*n* = 6, *p* < 0.001), indicating that there was on-target activity as observed in preclinical models. The baseline tumor sample from patient 11 with long-term survival (17.1 months) had significantly less baseline expression of CDK4/6-regulated genes than other baseline patient samples (*n* = 5, *p* = 2.8 × 10^−18^ using Student's paired *t*-test).

Paired pre- and on-treatment tumor biopsy samples were evaluable in six patients (50% yield). The baseline expression of 91 CDK4/6-regulated genes was significantly lower in patient 11 than in the other 5 patients (Fig. 4).

## Discussion

This is the first study to evaluate coinhibition of CDK4/6 and mTOR in patients with PC. Standard therapies for mPC are FOLFIRINOX (folinic acid, 5-fluorouracil, irinotecan, and oxaliplatin), gemcitabine plus nab-paclitaxel, and 5-fluorouracil, leucovorin, and nanoliposomal irinotecan.^21–23^ Patients who have disease progression on standard chemotherapy have limited treatment options outside of clinical trials. Previous studies of third-line therapy in mPC are limited but reveal stable disease in 25–31% of patients with no partial or complete responses (31% of patients in the combination GVAX/CRS-207 vaccine study had stable disease, but only 52% of these patients were treated in the third-line setting^24,25^). Likewise, a third-line Japanese study of paclitaxel in patients with prior disease progression on gemcitabine and S-1 resulted in a 40% stable disease rate with no objective responses seen.^26^

In patient-derived xenografts, we previously found that CDK4/6 inhibition was highly effective at limiting tumor growth, and had a profound impact on the proliferative index.^13^ This finding was also recapitulated in the analysis of primary tumor explants. Both of these patient-derived models recapitulate the histology and tumor microenvironment of PC. These data suggest that the vast majority of primary PC have the capacity to respond to CDK4/6 inhibition. However, although some preclinical models respond very effectively, others can develop resistance to CDK4/6 inhibition despite the loss of *CDKN2A*. In patients with *CDKN2A*-altered PC and biliary cancer enrolled on The Targeted Agent and Profiling Utilization Registry Study, there was limited clinical efficacy of CDK4/6 inhibitor monotherapy using palbociclib (no clinical responses or stable disease).^27^ We explored the mechanisms of resistance and found that there is a deregulation of parallel pathways that facilitate the bypass that involves signaling to other CDK/cyclins and involves the mTOR pathway.^13,14,28^ In drug screening, our group and others found that mTOR inhibitors were particularly potent cooperative agents with CDK4/6 inhibitors across all models^14,28^ and served to broaden the efficacy of CDK4/6 inhibition into resistant models.

Ribociclib and everolimus were well tolerated by our patient population, which was made up of heavily pretreated individuals due to their previous progression on both gemcitabine- and 5-fluorouracil-based chemotherapy. Hematological adverse events were common; notably, 33% of patients developed grade 3/4 neutropenia. There were no significant differences between adverse event rates between the two dose levels of ribociclib.

The best response seen was stable disease at 8 weeks in two patients (17%). Unfortunately, all other evaluable patients had disease progression as the best response. This finding was anticipated given the cytostatic nature of combined CDK4/6 and mTOR inhibition. Interestingly, three patients had an initial reduction in serum CA19-9, suggestive of potential biological efficacy. A planned phase II portion of the study to further assess efficacy was not pursued due to slow accrual.

We were able to demonstrate a reduction in CDK4/6-regulated gene expression in six patients with evaluable paired tumor biopsy samples. Patient 11 had relatively prolonged survival and significantly lower baseline CDK4/6-regulated gene expression than other patients, which appeared more prognostic than predictive of response to CDK4/6 inhibition. These findings concur with preclinical xenograft models and suggest that inhibiting CDK4/6 and mTOR can downregulate tumor CDK4/6 expression in patients with mPC.

This study illustrates the challenges of conducting clinical trials in third-line mPC. There was difficulty accruing patients whose disease had already progressed on multiple lines of therapy for mPC. Correlative studies were limited as only half of the patients had evaluable paired tumor biopsy samples. Efficacy could not be fully evaluated due to poor accrual.

## Conclusion

Although the combination has a favorable safety profile, ribociclib and everolimus should not be further studied in this treatment setting. Future study could examine the combination of CDK4/6 and mTOR inhibition with cytotoxic agents that have a lower risk of myelosuppression (e.g., cisplatin) or other targeted agents.

## Supplementary Material

Supplemental data

## Abbreviations Used

AEs: adverse events
ALT: alanine aminotransferase
AST: aspartate aminotransferase
CDKs: cyclin-dependent kinases
CTCAE: Common Terminology Criteria for Adverse Events
DLT: dose-limiting toxicity
ECG QT: (electrocardiogram) QT interval
ECOG PS: Eastern Cooperative Oncology Group Performance Status
FDA: U.S. Food and Drug Administration
KRAS: Kirsten rat sarcoma virus gene
mPC: metastatic pancreatic adenocarcinoma
mPFS: median progression-free survival
mOS: median overall survival
mTOR: mammalian target of rapamycin
PDX: patient-derived xenograft
PI3K: phosphatidylinositol 3-kinase
RB: retinoblastoma
RECIST: response evaluation criteria in solid tumors
RP2D: recommended phase II dose
TCGA: The Cancer Genome Atlas
