# Supplementary material for: A Phase I Study of Ribociclib Plus Everolimus in Patients with Metastatic Pancreatic Adenocarcinoma Refractory to Chemotherapy
Source: J Pancreat Cancer. 2020 Jun 22;6(1):45–54. doi: 10.1089/pancan.2020.0005 (PMC7337242; doi:10.1089/pancan.2020.0005)
Supplement: Supplemental data [file Supp_Table1.pdf]

Supplementary Data

Supplementary Table S1. Defining Dose-Limiting Toxicities Related to Ribociclib or Everolimus

Toxicity	DLT (per CTCAE v. 4.03)
Hematology	≥Grade 4 neutropenia (absolute neutrophil count <500/μL) lasting ≥7 consecutive days<br>≥Grade 4 thrombocytopenia<br>Grade 3 thrombocytopenia with clinically significant bleeding<br>Grade 3 or 4 febrile neutropenia
ECG QT interval	QTc interval ≥501 ms on at least two separate ECGs
Cardiac	≥Grade 3 cardiac toxicity, clinical signs of cardiac disease such as unstable angina, myocardial infarction, or troponin ≥grade 3
Gastrointestinal	≥Grade 3 vomiting ≥48 h despite optimal antiemetic therapy<br>≥Grade 3 diarrhea ≥48 h despite optimal antidiarrheal therapy<br>(Optimal therapy for vomiting and diarrhea should be based on institutional guidelines with consideration of the prohibited medications listed in these protocol guidelines).
Hepatobiliary	≥Grade 2 total bilirubin >7 consecutive days<br>≥Grade 3 total bilirubin<br>≥Grade 2 ALT with a ≥grade 2 bilirubin elevation of any duration in the absence of liver metastases<br>≥Grade 3 ALT for >4 consecutive days<br>Grade 4 ALT or AST<br>Grade 4 serum alkaline phosphatase >7 consecutive days
Renal	≥Grade 3 serum creatinine
Stomatitis	≥Grade 3—everolimus is associated with stomatitis. Per the Afinitor® package insert, topical treatments are recommended, but alcohol-, hydrogen peroxide-, iodine-, or thyme-containing mouthwashes should be avoided as they may exacerbate the condition. Antifungal agents should not be used unless fungal infection has been diagnosed.
Pneumonitis	Noninfectious pneumonitis—everolimus is associated with pneumonitis. The use of corticosteroids is suggested for grade 1–2 pneumonitis, recommended for grade 3 pneumonitis, and grade 4 pneumonitis should result in the cessation of everolimus.
Nonhematological nonhepatic adverse events	≥Grade 3 except for the following exceptions:
Exceptions to DLT criteria	Grade 3 alopecia<br><5 days of grade 3 fatigue<br>Grade 3 fever or infection without neutropenia <5 days duration<br>Grade 3 laboratory abnormalities that are responsive to oral supplementation or deemed by the investigator to be clinically insignificant

ALT, alanine aminotransferase; AST, aspartate aminotransferase; CTCAE, Common Terminology Criteria for Adverse Events; DLT, dose-limiting toxicity; ECG QT, (electrocardiogram) QT interval.
